# The moderating impact of neighborhood walkability on mHealth interventions to increase moderate to vigorous physical activity for insufficiently active adults in a randomized trial

**DOI:** 10.1186/s12966-023-01494-2

**Published:** 2023-08-15

**Authors:** Mindy L. McEntee, Jane C. Hurley, Christine B. Phillips, Steven P. Hooker, Michael Todd, Lawrence D. Frank, Marc A. Adams

**Affiliations:** 1 College of Health Solutions, Arizona State University, 425 North 5th Street, MC9020, Phoenix, AZ 85004 USA; 2 Department of Psychology, Clemson University, Clemson, SC USA; 3 College of Health and Human Services, San Diego State University, San Diego, CA USA; 4 College of Nursing and Health Innovations, Arizona State University, Phoenix, AZ USA; 5 Department of Urban Studies and Planning, University of California San Diego, San Diego, CA USA

**Keywords:** Physical activity, Moderate-to-vigorous physical activity, mHealth intervention, Ecological models, Neighborhood walkability

## Abstract

**Background:**

Ecological models suggest that interventions targeting specific behaviors are most effective when supported by the environment. This study prospectively examined the interactions between neighborhood walkability and an mHealth intervention in a large-scale, adequately powered trial to increase moderate-to-vigorous physical activity (MVPA).

**Methods:**

Healthy, insufficiently active adults (N = 512) were recruited purposefully from census block groups ranked on walkability (high/low) and socioeconomic status (SES, high/low). Participants were block-randomized in groups of four to WalkIT Arizona, a 12-month, 2 × 2 factorial trial evaluating adaptive versus static goal setting and immediate versus delayed financial reinforcement delivered via text messages. Participants wore ActiGraph GT9X accelerometers daily for one year. After recruitment, a walkability index was calculated uniquely for every participant using a 500-m street network buffer. Generalized linear mixed-effects hurdle models tested for interactions between walkability, intervention components, and phase (baseline vs. intervention) on: (1) likelihood of any (versus no) MVPA and (2) daily MVPA minutes, after adjusting for accelerometer wear time, neighborhood SES, and calendar month. Neighborhood walkability was probed at 5th, 25th, 50th, 75th, and 95th percentiles to explore the full range of effects.

**Results:**

Adaptive goal setting was more effective in increasing the likelihood of any MVPA and daily MVPA minutes, especially in lower walkable neighborhoods, while the magnitude of intervention effect declined as walkability increased. Immediate reinforcement showed a greater increase in any and daily MVPA compared to delayed reinforcement, especially relatively greater in higher walkable neighborhoods.

**Conclusions:**

Results partially supported the synergy hypotheses between neighborhood walkability and PA interventions and suggest the potential of tailoring interventions to individuals’ neighborhood characteristics.

**Trial Registration:**

Preregistered at clinicaltrials.gov (NCT02717663).

**Supplementary Information:**

The online version contains supplementary material available at 10.1186/s12966-023-01494-2.

## Introduction

Only a small percentage of U.S. adults meet national physical activity (PA) guidelines based on objective measures [1, 2], with minor improvements observed in population-level reported PA over the past two decades [3]. Ecological models of behavior change [4, 5] propose that interventions targeting individual behavior change are expected to be more effective when built environments, along with social and policy environments, support the target behavior [5, 6]. Numerous observational studies support this observation and have consistently shown PA appears sensitive to the design of built environments [4, 5, 7, 8]. The U.S. Community Preventive Services Task Force recommends built environment approaches to increase PA [9], such as using interventions that enable a person to respond to specific activity/inactivity-promoting environments. Understanding how the built environment *interacts* with PA interventions and programs is critical, as implementation of individual-level interventions or changes to the built environment alone are likely insufficient to increase PA substantially [10].

A recent narrative review of studies that examined how neighborhood environments interact with individual-level PA interventions found mixed results [11]. Although some studies found objectively-measured walkability moderated PA intervention effects [12–16], the direction has been inconsistent. For example, Zenk et al. [16] explored interactions between the Women’s Walking Program for African American women that included PA-focused workshops held at community health centers over 12 months and objective measures of walkability. They found no interaction between walkability and device-validated walking adherence. Alternatively, Colom et al. [14] explored interactions with objectively-measured walkability in the PREvención con DIeta MEDiterránea (PREDIMED)-Plus trial, a 12-month diet and PA education-based PA intervention for older men and women. Colom el al. found a standard deviation increase in walkability was associated with a change of 6 min of MVPA/day measured by accelerometer for their intervention group only, while the control group experienced no difference by walkability. By contrast, Kerr and colleagues [12] unexpectedly found that overweight men who completed a web-based PA and dietary behavior intervention and lived in objectively-measured *low* walkable neighborhoods experienced greater changes to their reported walking than those who lived in high walkable neighborhoods.

Notably, studies examining interactions between the objectively-measured built environment and physical activity have either been mainly retrospective, cross-sectional, or prospective observational studies [17, 18]. The few PA studies that have explored the moderating influences of built environments on individual-level interventions relied on retrospective measures of walkability (i.e., after study completion), and, as a result, were post hoc analyses [12, 19]. Consequently, published studies examining these potential moderating influences did not ensure sufficient variability in built environment features (e.g., samples comprised of low-to-moderately walkable neighborhoods mirroring what is found in most U.S. cities), or the studies were never designed and powered to test hypotheses for interactions between these interventions and walkability. Thus, previous studies have been exploratory, likely underpowered, and have presumably lacked variability in built environment features needed to capture meaningful differences in neighborhood walkability.

At least three challenges have made testing for potentiation between the built environment and individual-level PA interventions difficult. First, one ideally needs to observe that the intervention effectively increases PA. Second, the study needs to be designed a priori to ensure sufficient range (i.e., variability) in the built environment measures. Because randomizing participants to neighborhoods would not be possible, many observational studies recruited participants based on levels of walkability, which have had much better success in showing the effects of urban form on physical activity than convenience samples [8, 20]. Third, studies need to be sufficiently powered to test for the hypothesized interactions between environmental context and an intervention on PA adoption and maintenance.

The current study addresses many of these issues. We previously reported on the efficacy of an mHealth intervention’s components for improving free-living MVPA [21]. We found that participants receiving adaptive goals were more likely to engage in any (versus no) daily MVPA over a year, while participants receiving immediate reinforcement showed a greater increase in daily MVPA duration compared to those with delayed reinforcement. Joint effects of intervention types were also observed. Combined adaptive goals and immediate reinforcement yielded a significantly greater increase in the daily amount of MVPA compared to either delayed reinforcement group. Numerous mHealth interventions for PA, such as ours, have successfully targeted theoretical mechanisms and improved PA outcomes [22]. Secondly, and importantly, our recruitment efforts targeted and enrolled participants from high and low walkable census block-group areas identified before intervention enrollment to ensure adequate variability in built environment features across participants [23]. Finally, the current study was powered to test to detect interactions between neighborhood walkability and two intervention components (goal and reinforcement type) for PA adoption and maintenance, after accounting for attrition. Therefore, the current analysis was a registered primary aim of the study and reports on the cross-level interactions between individual-level interventions for MVPA and neighborhood walkability. We hypothesized, based on tenets of ecological models, that the intervention arms with the strongest overall effects for changing MVPA would show stronger effects in neighborhoods with higher walkability [5, 6]. This research addresses a major gap in the current literature on PA behavior change.

## Methods

### Study design

A detailed description of the study design and methods are presented elsewhere [23]. Briefly, inactive, healthy adults from Maricopa County, Arizona, were recruited between 2016 and 2018 into a year-long PA intervention called WalkIT Arizona (**Walk**ing **I**nterventions Through **T**exting in **Arizona**). GIS-measured walkability was calculated prior to the start of recruitment for all census block groups in the region (see [20]), which were classified as either higher (7th -10th deciles) or lower (1st -4th deciles) walkability areas. Block groups were also classified according to median annual household income from the American Community Survey as either higher (7th -10th deciles) or lower (1st -5th deciles) SES. The crossing of these classifications yielded a sampling design with four strata (i.e., higher walkability/higher SES, higher walkability/lower SES, lower walkability/higher SES, lower walkability/lower SES) which ensured variability in built environment characteristics while mitigating any potential confounding among block group SES and walkability and our PA interventions.

Eligible participants were computer block randomized (block size = 4) by stratum into one of four treatment groups for a 12-month intervention to evaluate the independent and joint effects of a 2 (adaptive versus static goal setting) x 2 (immediate versus delayed reinforcement) factorial trial to increase accelerometer-measured MVPA. As such, the factorial intervention was nested within an observational design of the built environment to evaluate interactions with walkability on MVPA. This study was approved by the institutional review board at Arizona State University and was prospectively registered with ClinicalTrials.gov (NCT02717663).

### Participants

Participants were 512 healthy, insufficiently active adults aged 18–60 living in eligible neighborhoods, willing to wear a wrist-worn accelerometer and receive daily text messages as part of an mHealth intervention to increase MVPA. Exclusion criteria included history of heart failure, type 2 diabetes, myocardial infarction, contraindications to exercise testing, currently or planning to become pregnant over the course of the study, and participation in other weight loss, diet, or PA programs. Those who planned to move or spend more than 30 days outside the area during the study were also excluded from participation. Insufficiently active status was initially assessed using the International Physical Activity Questionnaire (IPAQ) short form and subsequently confirmed with accelerometry (< 150 min/week of MVPA) during the 9-day baseline. This threshold of activity was chosen as it aligns with national classifications. All participants provided written informed consent and were compensated for completion of baseline ($20) and 12-month ($40) study measures. Participants and investigators were blinded to accumulated MVPA during baseline only; participants received feedback via text on their activity during the intervention based on their assigned intervention group.

### Intervention components

Participants were randomized into to one of four PA intervention groups, reflecting all possible combinations the 2 (adaptive vs. static goals) x 2 (immediate vs. delayed reinforcement) factorial design. All groups received an mHealth computer-automated intervention including: a single dose of educational materials on the first intervention day, daily text messages with MVPA goals, and performance feedback with financial reinforcement throughout the 12-month intervention. Intervention components are described below.

#### Goal setting

All participants received MVPA goals via text message after each successful sync of the accelerometer with the study server. Those randomized to static goals were prescribed 30 min of MVPA on 5 or more days/week (e.g., texted “Goal for 11/4 is 30 min”), consistent with federal PA guidelines [24]. Participants randomized to adaptive goals were informed their MVPA goal may increase, decrease, or stay the same each day, depending on their recent activity. Adaptive goals were calculated across a moving nine-day window reflecting each individual’s recent and unique activity using a 60th percentile algorithm, which was tested in prior studies [25–27]. For example, a participant with recent MVPA durations (rank ordered) of 5, 7, 9, 9, 11, 14, 15, 17, and 20 min/day, the 60th rank-percentile would yield a goal of 14 min/day for the next day (e.g., “Goal for 11/15 is 14 min.“).

#### Reinforcement timing

All participants received praise feedback from a pool of text messages for meeting daily goals. Participants received a simple confirmation message when they did not meet a daily goal to avoid discouragement (e.g., “Sync Received. 3 min. Goal for 11/16 is 13 min”). Participants were informed they had the opportunity to earn financial micro-incentives at the start of the intervention. Based on prior studies [25, 26], it was anticipated participants would meet 40–73% of their daily goals over the course of the 12-month intervention, or $156 to $265 in incentives. Those in the immediate reinforcement condition earned points each day they met their MVPA goal (100 points = $1, averaging $1 each day goals were met), which was automatically cashed out as an e-gift card once the participant earned $5.00. Participants could choose from a number of popular retailers via the mHealth system and change at any time. To encourage participation among those randomized to delayed reinforcement, e-gift card payment was paid every 60 days on an escalating scale (i.e., $15 in month 2 [M2], $30 in M4, $50 in M6, $75 in M8, and $95 in M10) for sufficient wear (4 of last 7 days) and recently syncing the accelerometer. This amount was selected to match the average maximum incentive earned by participants in the immediate reinforcement condition.

### Measures

#### Moderate to vigorous physical activity (MVPA)

Objective PA was measured via a small wrist-worn accelerometer, the ActiGraph GT9X Link (ActiGraph, LLC, Pensacola, FL, USA). During the yearlong intervention, participants were asked to wear the accelerometer for at least 10 hours daily (instructed to remove the device during bathing/swimming/contact sports). Vector magnitude (VM) was calculated at 1-minute epoch intervals for vertical, antero-posterior, and medio-lateral planes. An individualized VM counts/minute threshold for MVPA was calibrated for each participant at baseline via breath-by-breath indirect calorimetry during a continuous treadmill walk test at speeds of 2, 3, and 4 mph. MVPA was defined by meeting or exceeding the individualized VM threshold (intensity > 3 METS) and GT9X step count for the corresponding 1-minute epoch ≥ 30 [23]. Non-wear was determined using the Choi algorithm, defined as ≥ 90 consecutive zero counts per minute with ≤ 2 minutes of non-zeros on the vertical axis [28]. At least 6 hours of wear or achievement of a daily PA goal were required for that day’s data to be included in these analyses.

#### Individual-level Walkability Index

Participant addresses were geocoded using ArcGIS 10.5 software (ESRI, Redlands, CA) using the U.S. Census Tigerline address feature. ArcGIS Network Analyst was used to develop a 500 m street-network buffers around participants’ home addresses, which is consistent with other international studies [29], as consensus on buffer size does not exist [30, 31]. Within each participant’s buffer, residential density (i.e., residential units / residential land area in buffer), land use mix (entropy of several land uses including residential, retail, entertainment, civic, food, and recreational in buffer area), street network connectivity (# of intersections / buffer area), and density of public transit access (i.e., number of transit stops and stations / buffer area) were calculated. Individual-level walkability index was computed by summing each of these component z-scores [20].

#### Covariates

All models were adjusted for daily accelerometer wear time (centered), block group SES, and calendar month. The method for determining daily wear time was described above. Calendar month was included to account for potential effects of extreme summer temperatures on outdoor activity.

### Statistical analyses

An intent-to-treat approach analyzed participants by their randomized assignment to components. Generalized linear mixed models were used to examine main and joint effects of individual-level neighborhood walkability, intervention components (goal type, reinforcement timing), and phase (baseline vs. intervention). Further model specification was guided by the distribution of outcome data. MVPA bout minutes were positively skewed with a relatively large number of zero values. Because of the inflated number of daily zero-minute values over the year-long intervention, we selected a two-part hurdle modeling approach over zero-inflation models, with zero values reflecting physical inactivity during accelerometer wear time. Accordingly, each analysis comprised two parts: (1) a mixed effects binary logit model to estimate probability of engaging in any (versus no) MVPA each day, and (2) a mixed effects negative binomial regression model to estimate MVPA bout minutes/day on days participants were active.

Models were estimated in R with the glmmTMB package [32] specifying a zero-truncated negative binomial error distribution (family = truncate_nbinom2). Intervention components (goal type, reinforcement timing) were represented with effect coded vectors. Neighborhood walkability, intervention components, and phase were entered as fixed effect terms along with random participant-level intercepts. Interactions reflected the joint effects of individual-level neighborhood walkability on intervention components (modeled separately) by phase (average baseline and intervention phases), controlling for accelerometer wear time (centered), block group SES (high vs. low), and calendar month. Effect sizes for model summaries are reported: odd ratios (*OR*) for binary logit models and risk ratios (*RR*) for negative binomial count models. *OR*, *RR*, and 95% confidence intervals (95% CI) are the exponentiated coefficients of conditional estimates from R model output. The effects package was used to visualize interactions [33, 34]. We pre-specified probing intervention effects at the 5th, 25th, 50th, 75th, and 95th percentiles of walkability scores to examine effects across the range of individual neighborhood walkability. A priori power analyses indicated a sample size of 480 afforded 0.80 power to detect interaction effects corresponding to a 4.2 min/day difference in differences (i.e., for lower vs. higher walkable, a 4.2-min/day between-neighborhood difference in the magnitude of the intervention component (main effects) at 12 months) for MVPA.

## Results

**Fig. 1:**
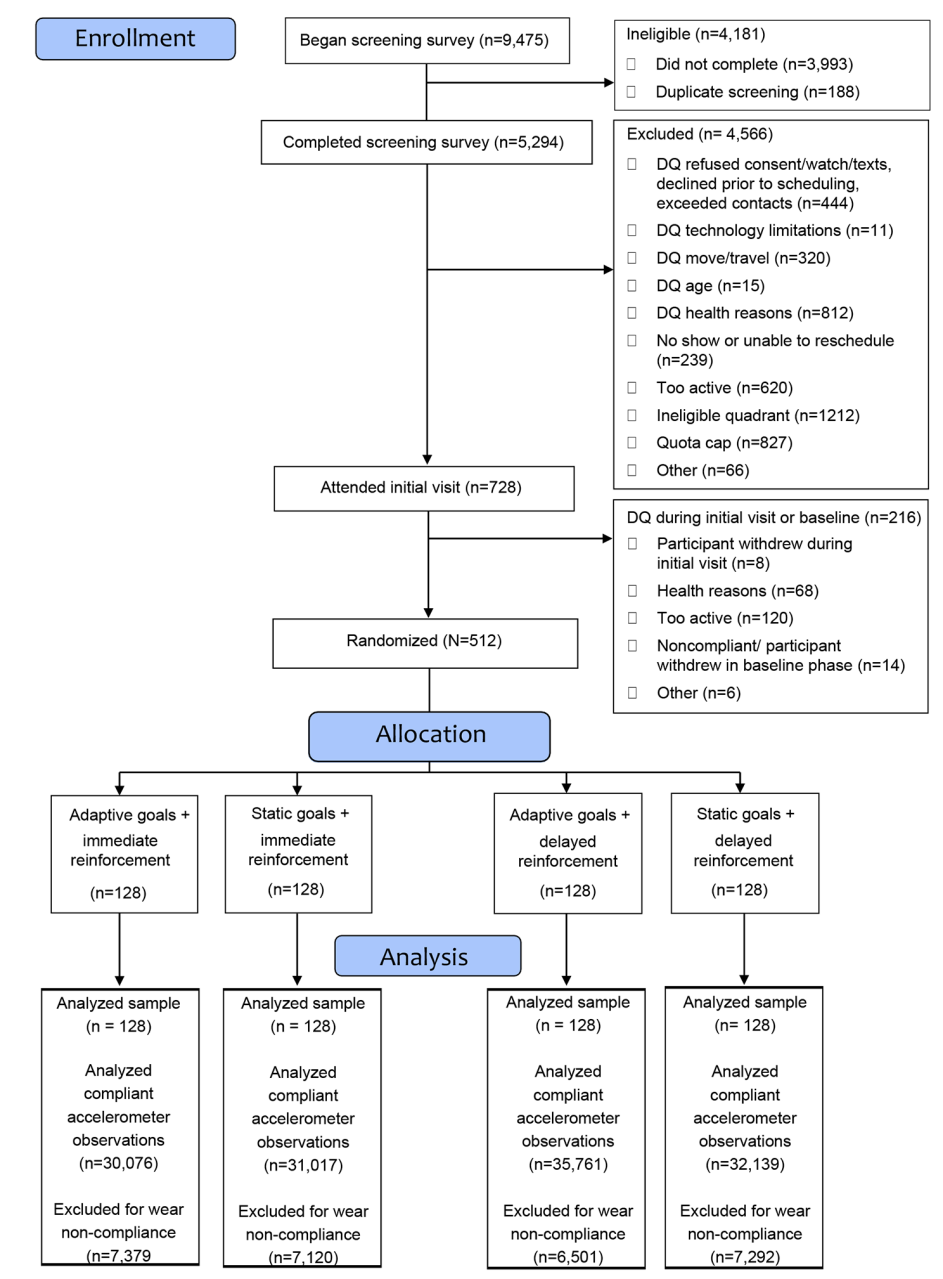
WalkIT Arizona Participant CONSORT Diagram

The CONSORT diagram is depicted in Fig. 1. Participants were 64.3% female, 18.5% Hispanic, 6.2% African American, and 83% White. They averaged 45.3 years of age (SD = 9.2), with a mean BMI of 33.1 (SD = 7.1). Demographics and participant characteristics are displayed intervention group in Table 1. Primary intervention outcomes are reported elsewhere [21]. Appendix 1 shows that participants’ 500-meter walkability index values ranged from a z-score of -6.46 to + 10.1 (mean = 0.02, standard deviation = 2.46).

**Table 1:** Participant characteristics by intervention group

	Total (N = 512)	Adaptive Goal + Immediate Reinforcement (n = 128)	Static Goal + Immediate Reinforcement (n = 128)	Adaptive Goal + Delayed Reinforcement (n = 128)	Static Goal + Delayed Reinforcement (n = 128)
Female, %	64.3	64.1	62.5	63.3	68.0
Age, Mean (SD)	45.5 (9.1)	45.6 (9.5)	46.0 (8.9)	46.7 (8.6)	43.5 (9.3)
Race and ethnicity					
Caucasian or white, %	84.0	84.4	82.8	82.0	82.8
African American or Black, %	6.3	3.9	7.0	7.0	6.3
American Indian or Alaskan Native, %	2.7	3.1	2.3	1.6	3.9
Asian, %	2.3	3.1	2.3	2.3	1.6
Native Hawaiian or other Pacific Islander, %	1.4	2.3	0.8	1.6	0.8
Prefer not to answer, %	6.2	3.8	6.2	7.8	7.0
Hispanic or Latino, %	18.8	17.2	20.3	18.8	18.8
BMI, Mean (SD)	33.9 (7.3)	33.7 (7.3)	33.8 (7.3)	33.6 (7.0)	34.5 (7.6)
Current tobacco smoker, %	5.0	2.4	7.8	3.9	6.3
Current E-smoker, %	2.0	1.6	2.4	0.8	3.2
Married/living with partner, %	67.5	64.1	66.4	72.7	67.2
Residence type, %					
Single family house	76.1	72.7	71.5	81.3	78.9
Apartment	13.3	12.9	15.4	11.7	13.3
Years at current residence, mean (SD)	7.3 (7.4)	7.3 (7.8)	7.2 (7.5)	8.2 (7.0)	6.4 (7.1)
Has children in household, %	49.1	47.7	47.5	50.0	50.8
# of children in household, median	0	0	0	0.5	1
Household income, median	$60,000–79,999	$80,000–99,999	$60,000–79,999	$60,000–79,999	$80,000- 99,999
Education, median	College graduate	College graduate	College graduate	College graduate	College graduate
Employed, full time %	76.2	76.6	75.8	73.4	78.9
Distance from home to work (meters), median	16,316	15,368	16,718	15,597	16,926
Individual level walkability, median	-0.08	0.16	-0.22	-0.38	0.20

Note. Participants were asked to select “all that apply” for race/ethnicity, cumulative is > 100%

### Neighborhood walkability x goal x phase

**Fig. 2:**
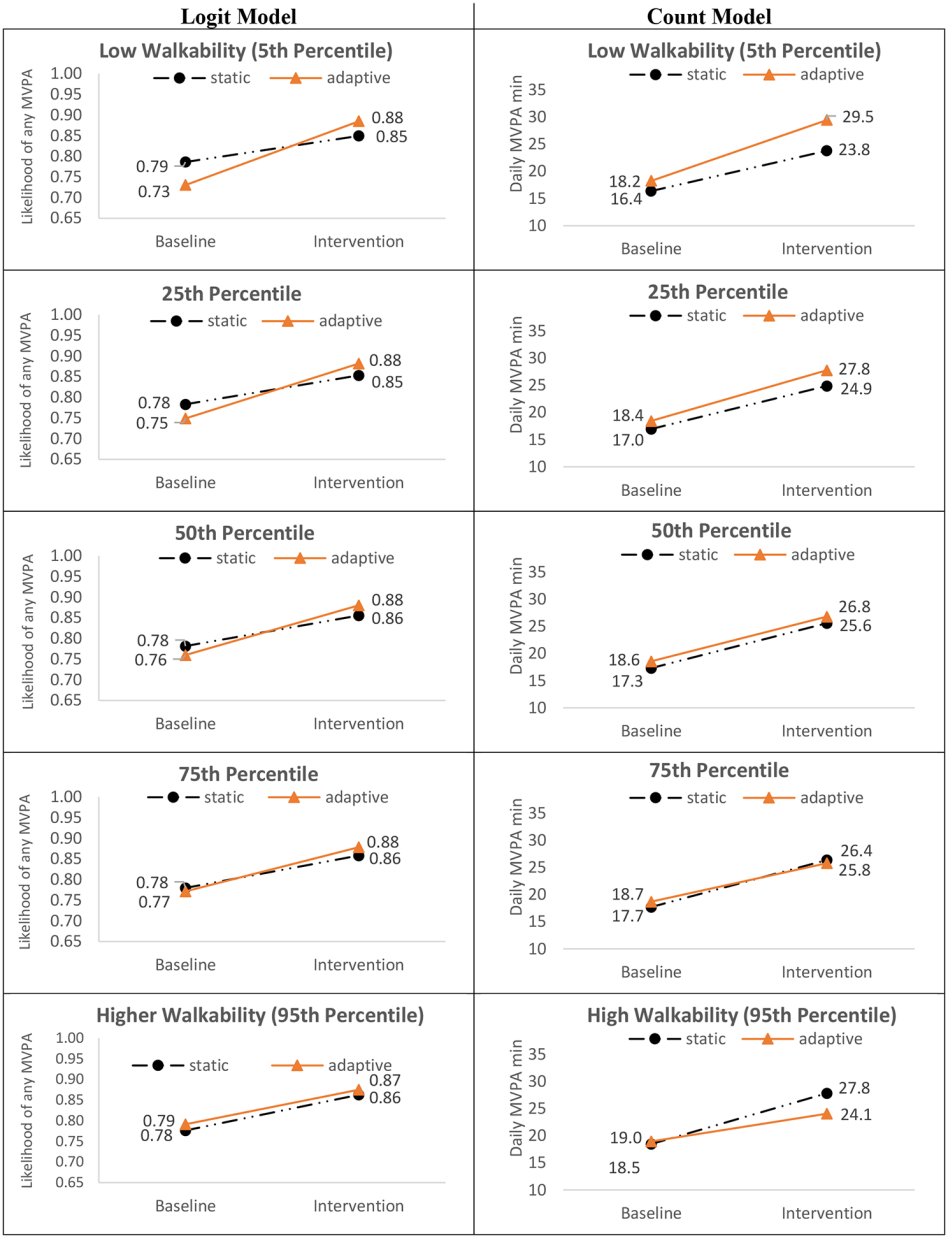
Probed Walkability x Goal Type x Phase Interactions (N = 512)

**Fig. 3:**
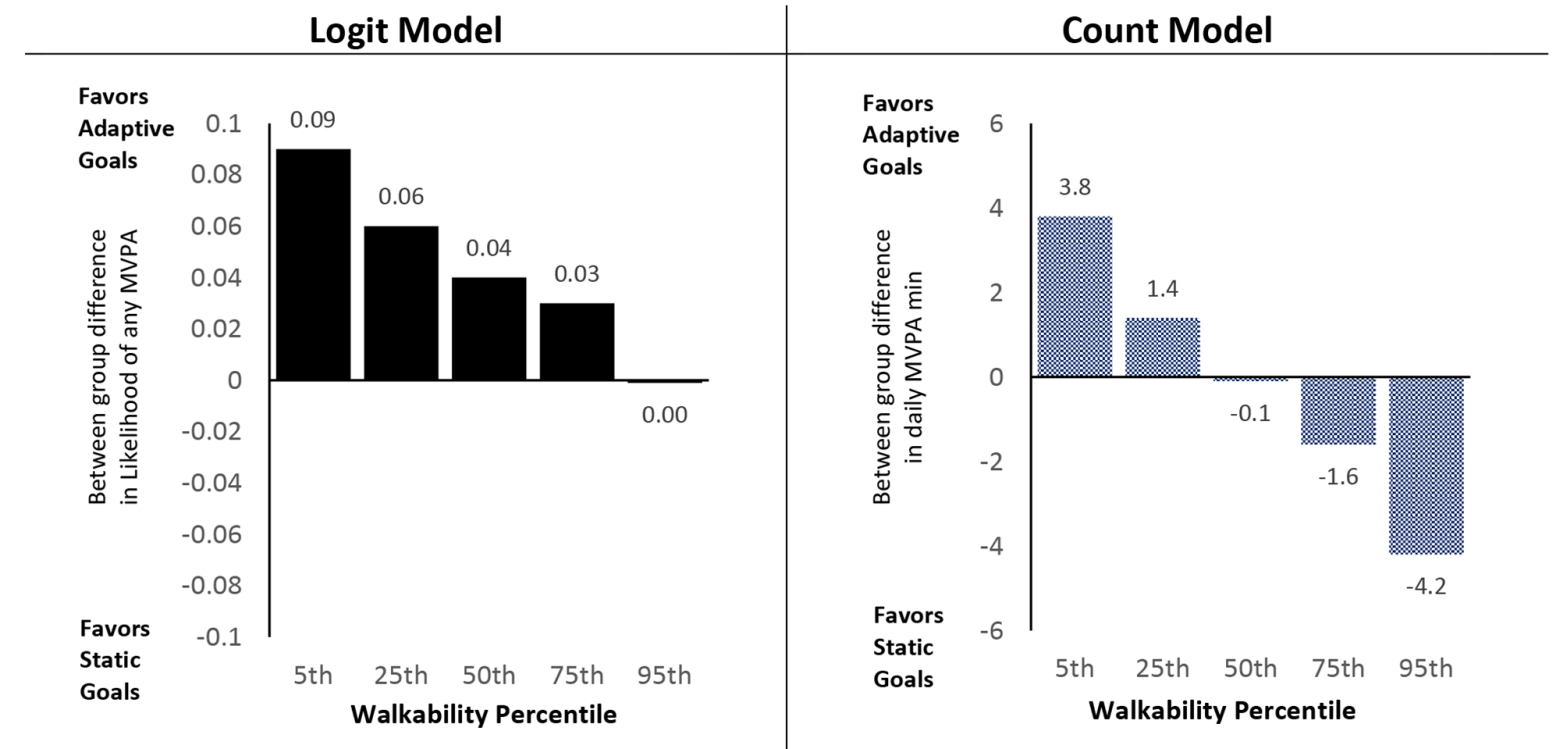
Walkability x Goal Type x Phase: Relative Differences in Goal Type Effect Across Walkability (N = 512)

Full model results are summarized in Table 2 and discussed below. Figure 2 shows the trajectory and magnitude of change from baseline to intervention for both goal-type groups at each of the probed levels of walkability. Figure 3 further summarizes the differences by goal type across probed walkability levels.

**Table 2:** Models for Walkability x Goal Type x Phase (N = 512)

	Logit Model		Count Model		
Parameter	OR (95% CI)	*P* value		RR (95% CI)	*P* value
Intercept	0.41 (0.33, 0.50)	< 0.001***		17.12 (15.94, 18.39)	< 0.001***
Wear time	0.9989 (0.9989, 0.9990)	< 0.001***		1.00009 (0.00007, 1.00011)	< 0.001***
SES (low)	0.89 (0.69, 1.14)	0.343		1.02 (0.93, 1.12)	0.670
Reinforcement Timing (delayed)	0.77 (0.60, 0.98)	0.034*		1.15 (1.05, 1.25)	0.002**
Goal Type (static)	1.13 (0.85, 1.49)	0.406		1.07 (0.97, 1.19)	0.178
Walkability	0.98 (0.93, 1.04)	0.574		1.01 (0.99, 1.03)	0.365
Phase (baseline)	0.51 (0.47, 0.55)	< 0.001***		1.46 (1.42, 1.50)	< 0.001***
Walkability x Goal	0.95 (0.85, 1.07)	0.411		0.99 (0.95, 1.03)	0.632
Goal x Phase	0.72 (0.62, 0.83)	< 0.001***		0.97 (0.92, 1.03)	0.317
Walkability x Phase	1.02 (0.98, 1.05)	0.313		0.988 (0.977, 0.998)	0.018*
Walkability x Goal x Phase	1.08 (1.01, 1.15)	0.023*		0.97 (0.95, 0.99)	0.001**

*Note*. Monthly effects excluded for simplicity. Referent groups for parameters listed in parentheses. Goal Type and Reinforcement Timing were independently effect coded (-0.5, 0.5) and Phase was dummy coded (0 = baseline,1 = intervention). OR = odds ratio, RR = risk ratio, CI = confidence interval. OR for the hurdle model reflects odds of any (vs. no) MVPA, RR for the count model reflects MVPA bout minutes/day on active days. OR, RR, and 95% CI are exponentiated coefficients of conditional estimates. .p < .1, *p < .05, **p < .01, ***p < .001

#### Likelihood of any MVPA

Across intervention groups, likelihood of engagement in any (versus no) daily MVPA increased from baseline (range: 0.73 to 0.79) to the intervention phase (range: 0.85 to 0.88). There was a significant Neighborhood Walkability x Goal Type x Phase interaction for the logit model (OR = 1.08, 95% CI: 1.01, 1.15, p = .023). As displayed in the left column of Fig. 2, participants randomized to adaptive goals showed a greater increase in the likelihood of any MVPA from baseline to intervention relative to static goals. The magnitude of this effect was strongest for those living in lower walkable neighborhoods and dissipated with greater levels of walkability until it disappeared at the 95th percentile, as depicted in the left panel of Fig. 3.

#### Daily MVPA bout minutes

Among participants who engaged in at least one daily bout of MVPA, model adjusted total MVPA bout minutes/day and walkability were positively associated at baseline; higher levels of walkability were associated with higher baseline MVPA levels for both groups. MVPA increased for all intervention groups from baseline (range: 16.4 to 19.0 min) to the intervention phase (range: 23.8 to 29.5 min). There was a significant Neighborhood Walkability x Goal Type x Phase interaction (OR = 0.97, 95% CI: 0.95, 0.99, p = .001). As depicted in the right column of Fig. 2, the intervention effects of goal type from baseline to intervention varied by walkability. For participants randomized to adaptive goals, those living in lower walkable neighborhoods experienced a larger increase in the duration of MVPA bout minutes/day from baseline to intervention relative to those living in higher walkable neighborhoods (+ 11.3 min/day for 5th percentile, + 9.4 min/day for 25th percentile, + 7.1 min/day for 75th percentile, + 5.1 min/day for 95th percentile). This pattern was reversed among those randomized to static goals, where those living in high walkable neighborhoods showed a greater increase in daily MVPA duration from baseline to intervention phase relative to those living in lower walkability areas (+ 9.3 min/day at 95th percentile, + 8.7 min/day at the 75th percentile, + 7.9 min/day for 25th percentile, + 7.4 min/day for 5th percentile). The right panel of Fig. 3 summarizes the goal type differences by levels walkability.

### Neighborhood walkability x reinforcement timing x phase

**Fig. 4:**
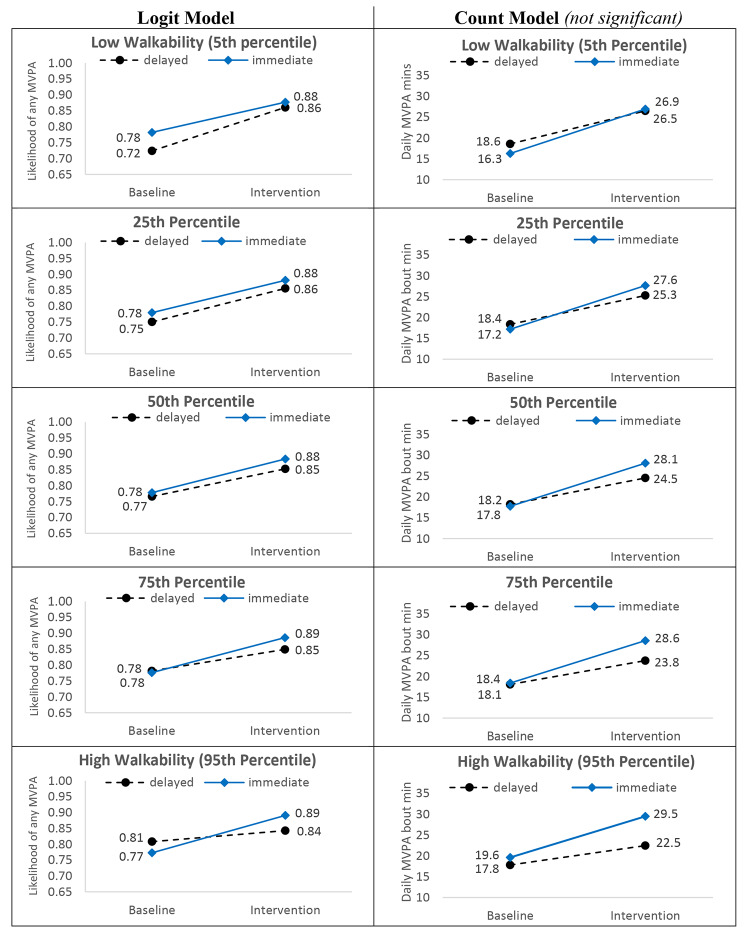
Probed Walkability x Reinforcement Timing x Phase Interactions (N = 512)

**Fig. 5:**
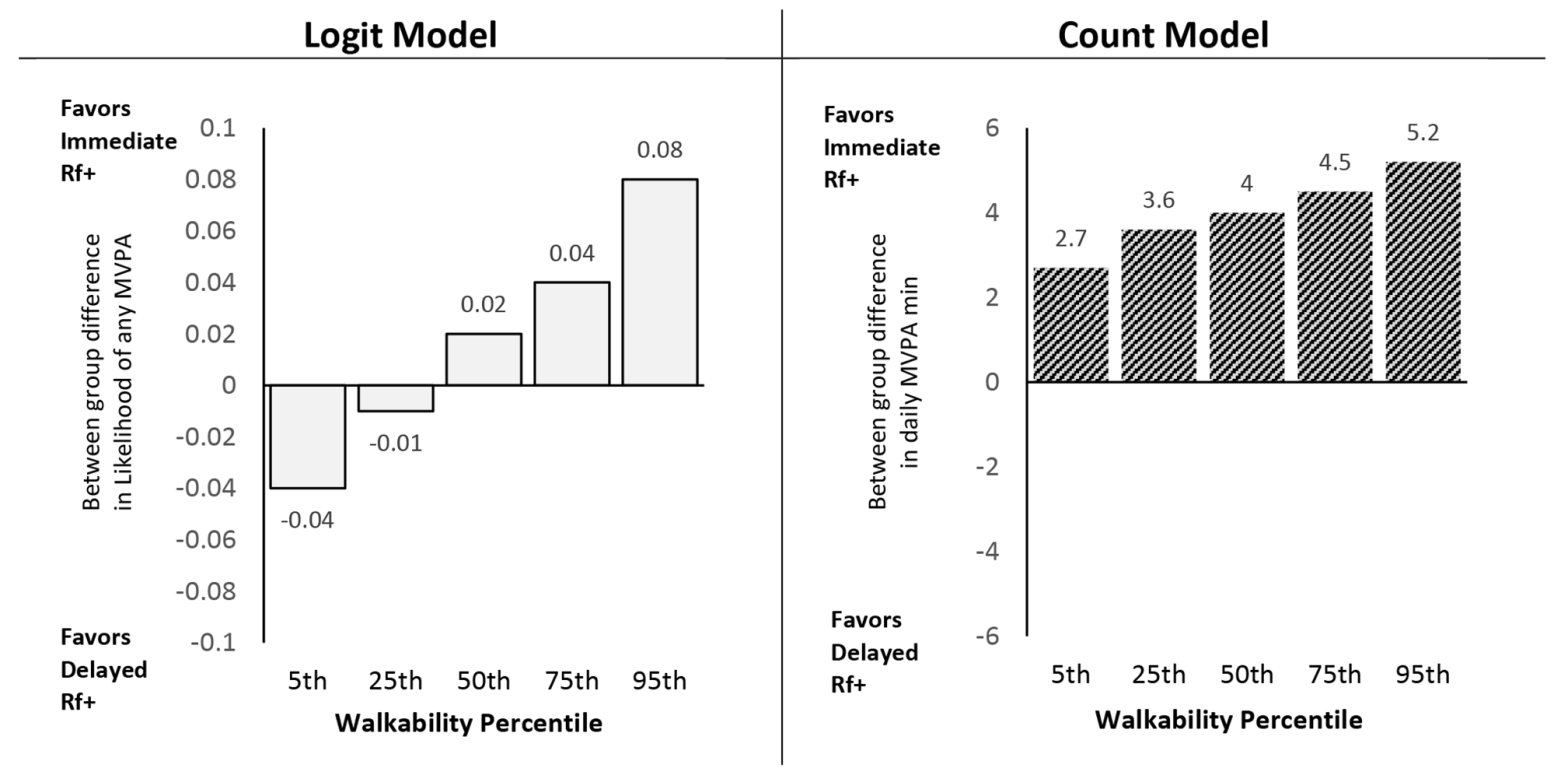
Walkability x Reinforcement Timing x Phase: Relative Differences in Reinforcement Timing Effect Across Walkability (N = 512)

Full model results are summarized in Table 3 and discussed below. Figure 4 shows the trajectory and magnitude of change from baseline to intervention for both reinforcement groups at each of the probed levels of neighborhood walkability. Figure 5 shows the differences in intervention effect by reinforcement timing across probed walkability levels.

**Table 3:** Models for Walkability x Reinforcement x Phase (N = 512)

	Logit Model			Count Model	
Parameter		OR (95% CI)	*P* value	RR (95% CI)	*P* value
Intercept		0.40 (0.33, 0.49)	< 0.001***	17.45 (16.25, 18.74)	< 0.001***
Wear time		0.9989 (0.9989, 0.9990)	< 0.001***	1.00008 (1.00007, 1.00010)	< 0.001***
SES (low)		0.89 (0.69, 1.14)	0.343	1.02 (0.93, 1.11)	0.717
Goal Type (static)		0.83 (0.65, 1.06)	0.146	1.04 (0.95, 1.13)	0.402
Reinforcement Timing (delayed)		0.94 (0.71, 1.24)	0.652	0.98 (0.88, 1.08)	0.642
Walkability		0.97 (0.92, 1.03)	0.393	1.01 (0.99, 1.03)	0.428
Phase (baseline)		0.51 (0.47, 0.55)	< 0.001***	1.46 (1.42, 1.50)	< 0.001***
Walkability x Reinforcement		1.07 (0.95, 1.20)	0.274	1.03 (0.99, 1.07)	0.184
Reinforcement x Phase		0.81 (0.70, 0.94)	0.006**	1.18 (1.12, 1.24)	< 0.001***
Walkability x Phase		1.03 (0.99, 1.06)	0.104	0.987 (0.976, 0.997)	0.012*
Walkability x Reinf. x Phase		0.91 (0.85, 0.97)	0.002**	1.003 (0.98, 1.02)	0.756

*Note*. Monthly effects excluded for simplicity. Referent groups for parameters listed in parentheses. Goal Type and Reinforcement Timing were independently effect coded (-0.5, 0.5) and Phase was dummy coded (0 = baseline,1 = intervention). OR = odds ratio, RR = risk ratio, CI = confidence interval. OR for the hurdle model reflects odds of any (vs. no) MVPA, RR for the count model reflects MVPA bout minutes/day on active days. OR, RR, and 95% CI are exponentiated coefficients of conditional estimates. .p < .1, *p < .05, **p < .01, ***p < .001

#### Likelihood of any MVPA

Across all groups in the logit model, likelihood of engagement in any (versus no) daily MVPA increased from baseline (range: 0.72 to 0.81) to intervention (range: 0.84 to 0.89). There was a significant Neighborhood Walkability x Reinforcement Timing x Phase interaction (OR = 0.91, 95% CI: 0.85, 0.97, p = .002). As shown in the left column of Fig. 4, the magnitude of increase in likelihood of MVPA from baseline to intervention was largely consistent for participants randomized to immediate reinforcement across levels of neighborhood walkability (i.e., + 0.10 to + 0.12 across all walkability percentiles). Conversely, participants assigned to delayed reinforcement living in low walkable neighborhoods showed a stronger increase in the likelihood of any MVPA among participants relative to those with delayed rewards in higher walkable areas (0.14 for 5th percentile, + 0.11 for 25th percentile, + 0.07 for 75th percentile, + 0.03 for 95th percentile). Participants randomized to immediate reinforcement had a modestly greater likelihood of any MVPA during the intervention compared to those with delayed reinforcement for all levels of walkability, as indicated by the difference in intervention likelihood between shown in Fig. 4 (range: 0.02 to 0.04). Figure 5 (left panel) summarizes the reinforcement timing differences by levels of walkability.

#### Daily MVPA bout minutes

Among participants who engaged in at least one daily bout of MVPA, model adjusted total MVPA bout minutes/day increased across all groups from baseline (range: 16.3 to 19.6 min) to intervention (range: 22.5 to 29.5 min). The three-way Neighborhood Walkability x Reinforcement x Phase interaction was not significant. However, there was a significant Reinforcement x Phase interaction, RR = 1.18, 95% CI: 1.12, 1.24, p < .001, such that immediate reinforcement resulted in a greater increase in MVPA from baseline to intervention (+ 10.3 min/day) relative to delayed reinforcement (+ 6.3 min/day). There was also a significant Neighborhood Walkability x Phase interaction, RR = 0.987, 95% CI: 0.976, 0.997, p = .012, such that the magnitude of intervention effect (increase in daily MVPA duration) was greatest for those living in low walkable neighborhoods and declined as walkability increased: +9.3 min/day for 5th percentile, + 8.7 min/day for 25th percentile, + 8.2 min/day for 50th percentile, + 7.8 min/day for 75th percentile, + 7.0 min/day for 95th percentile. This progressive increase in daily MVPA duration for higher walkable neighborhoods is shown in the right column of Fig. 4 and right panel of Fig. 5.

## Discussion

Unlike previous studies, the current prospective trial was powered a priori to test for cross-level interactions between neighborhood walkability and individual-level behavioral intervention components for MVPA adoption among insufficiently active adults recruited from neighborhoods known to differ in levels of walkability. Thus, this trial went beyond single-level interventions and observational studies exploring these relationships independently for physical activity and found mixed results.

Our hypotheses were based on ecological theory for physical activity [6]. Contrary to our hypotheses, adaptive goals were associated with greater intervention effects for those in *lower* walkable neighborhoods (e.g., a greater increase in likelihood to engage in any MVPA and a larger increase in MVPA minutes/day from baseline to intervention). For the likelihood of *any* MVPA, the magnitude of difference between goal types decreased as walkability increased, though there was no level of walkability at which static goals outperformed adaptive goals. By contrast, adaptive goals were associated with increased MVPA minutes/day from baseline to intervention across all levels of walkability, and this effect increased as walkability decreased, while the static goal group increased MVPA min/day, and this effect became stronger with higher levels of walkability.

McCormack et al.’s review of the literature for built environment by intervention interactions [11] provides a helpful framework for discussing the patterns in this trial. Their review found mixed results in the literature with 75% of studies reflecting an “invariant” pattern (i.e., neither positive nor negative effect) between interventions and at least one environmental variable. The current results for the adaptive goal condition for the logit and count models reflect what McCormack et al. called a “compensation” pattern. In contrast, the static goal group reflected little differences across walkability levels, or an invariant pattern, for logit model, but an “amplification” or synergistic pattern for count model. The combination of interaction patterns by group (Fig. 3) suggests that an adaptive goal-setting intervention is most effective in and helps compensate for activity-unsupportive neighborhoods, but static goals perform better in more activity-supportive neighborhoods. Because many U.S. cities designed after the advent of the automobile reflect a predominantly car-centric, low-walkable design [35, 36], adaptive goal setting interventions should be considered a viable alternative to common “one-size-fits all” goal setting approaches (e.g., 30 min/day or 10,000 steps) until enhancements to walkability are the norm, or interventions can tailor the goal setting approach to an individual’s neighborhood type.

The results for reinforcement components differed for logit and count models. Participants in the immediate reinforcement condition were more likely to increase any MVPA (logit model) during the intervention phase than the delayed reinforcement group. This change for the immediate reinforcement condition was invariant across all levels of walkability. In contrast, for the delayed reinforcement condition, observed improvements in any MVPA decreased as walkability increased, suggesting a suppression pattern. The MVPA minutes/day effects (count model) reflected a similar pattern to the logit model. When considering the combined interaction patterns by group (Fig. 4), these results suggest immediate outperforms delayed reinforcement only in more activity-supportive neighborhoods, supporting our potentiation hypotheses. However, it is surprising to observe an invariant pattern within the immediate reinforcement condition across levels of walkability. Perhaps insufficiently active adults living in higher walkable neighborhoods represent a unique population, and these individuals may have particular circumstances (e.g., stronger work or life obligations, real or perceived safety concerns in more populated areas) that overcome the pull of their walkable neighborhood. As such, these individuals may require different or even stronger intervention components/stimuli to overcome their particular circumstances, while the delayed reinforcement intervention was strong enough to promote greater MVPA for insufficiently active individuals living in unsupportive, but not supportive, environments. More research is warranted among insufficiently active adults living in walkable neighborhoods to confirm and understand this result.

These mixed results and patterns are consistent with the current mixed literature [11]. However, given that the current study design and methods overcame many of the limitations highlighted in the literature, the results are surprising and suggest unaccounted-for factors or a need to refine ecological hypotheses in this area. Unaccounted-for micro-scale pedestrian environment features, such as traffic, safety, demographic characteristics, individual perceptions, or other social elements, may help explain the results above and beyond macro-scale environment features [13, 37–39]. While the current study focused on GIS-measured walkability and accelerometer-measured MVPA, even less research has examined features of the perceived built environment at the pedestrian streetscape level (e.g., sidewalk buffers, crime safety, esthetics, benches) on PA. The studies that have examined micro-scale features found only self-reported safety from traffic [12], traffic control devices, and crosswalks [13] interacted with individual-level PA interventions in women to increase reported physical activity. Such inconsistent results run counter to hypotheses that higher walkability should potentiate individual-level PA interventions across the general population and appear consistent with our results of compensation effects.

The current analyses expand on the main study outcomes by accounting for the macro-level setting of individual neighborhoods. In line with social-ecological theory, interactions between behavioral interventions and the built environment are crucial, as their effects are not mutually exclusive. Theory posits that interventions to improve population health are likely more effective when they address modifiable targets at multiple levels (e.g., individual behaviors + built environmental features + public policies) that facilitate behavior change [5]. This study provides novel insights regarding how the broader level of environmental influence (i.e., macro-level neighborhood walkability) interacts with individual-level intervention components to influence MVPA, highlighting the complex nature of interactions. Our findings do not support the notion of a unidirectional interaction between ecological levels leading to synergistic or potentiating effects of behavior changes interventions across different levels of walkability, indicating the need for further consideration of environmental factors or refinement of underspecified ecological hypotheses. Results may help guide tailoring decisions for individual-level interventions and/or identify groups most likely to respond to maximize return on investment costs.

While all intervention groups increased MVPA from baseline to intervention, there were similar upper limits of intervention effects across components. With logit models, average likelihood of any (vs. no) MVPA on a given day appeared to peak around 0.88 for both goal and reinforcement type. With the count models, average daily MVPA appeared to reach an upper limit around 29.5 min/day for both goal and reinforcement type. Further analyses may be helpful in identifying whether these limits more accurately reflect variability in individual activity levels day to day or variability in overall responsiveness to the intervention.

Several methodological considerations should be noted. Strengths of the current study included an evidence-based intervention that increased adults’ MVPA, a priori cross-level hypotheses, power sufficient to detect walkability by intervention component interactions, the use of objective measures of MVPA and built environment features, a factorial comparative effectiveness design to test intervention components, and sampling methods that ensure adequate variability in walkability, socioeconomic status, and weather before the intervention onset. There are a few key limitations worth considering. First, the WalkIT Arizona study took place in a sprawling U.S. metropolitan area with overall lower walkability than other international cities. While study methods intentionally sampled a broad range of walkability (Appendix 1), findings may not generalize to other geographic conditions, such as predominately high walkable cities or rural areas inside or outside the US. However, the use of standardized walkability z-scores facilitates comparison across cities and countries [29]. Second, calculations for individual neighborhood walkability were based on home addresses; we did not assess or otherwise account for walkability surrounding participants’ place of work or time-varying effects between interventions and walkability. Additional factors unaccounted for in these analyses included the lack of GPS measures to determine whether MVPA occurred inside or outside of participant nightborhoods and the lack of specific environment components such as parks, commute times, or micro-scale features such as sidewalks or crosswalks, etc.). Such factors could help explain the unexpected effects. Similarly, we did not adjust for the distance between participants’ home and work locations, which may be meaningful for those who commute via walking or biking. Finally, “phase” was used as a simplified measure of time (baseline vs. intervention) in analyses. This decision was based on efforts to maximize power and maintain interpretability, though our findings may not have captured important nuances in patterns of change over the course of the year-long intervention. Future research is needed to examine change over time in greater detail, including how neighborhood walkability predicts maintenance of MVPA following a successful intervention.

Conclusion. This study offers a novel analysis of the interplay between individual-level interventions to increase MVPA and variations in the built environment that either support or hinder physical activity. The findings highlight the complexity of investigating cross-level interactions as posited by ecological models. Results suggest that adaptive goal-setting could help counteract the negative effects of activity-unsupportive environments, whereas immediate rewards may be most effective in neighborhoods that are already supportive of physical activity. These current results offer evidence towards refining theory in light of the mixed findings. Further studies and refinement of ecological hypotheses may be necessary to advance the field.

### Electronic supplementary material

Below is the link to the electronic supplementary material.

Supplementary Material 1: WalkIT Arizona’ TIDieR - Intervention Description and Replication Checklist.

Supplementary Material 2: WalkIT Arizona CONSORT 2010 checklist.

Supplementary Material 3: Appendix Figure 1. Walkability Index scores for participants’ 500-m network buffers (N=512).

## Data Availability

The datasets used and/or analysed during the current study are available from the corresponding author on reasonable request pending IRB approvals.

## List of Abbreviations

PA: Physical activity
MVPA: Moderate-to-vigorous physical activity
Min/day: Minutes per day
SES: Socioeconomic status
OR: Odd ratios
RR: Risk ratios

## References

[1] Troiano RP, Berrigan D, Dodd KW, Masse LC, Tilert T, McDowell M (2008). Physical activity in the United States measured by accelerometer. Med Sci Sports Exerc.

[2] Tucker JM, Welk GJ, Beyler NK (2011). Physical activity in U.S.: adults compliance with the physical activity guidelines for Americans. Am J Prev Med.

[3] Du Y, Liu B, Sun Y, Snetselaar LG, Wallace RB, Bao W (2019). Trends in adherence to the physical activity guidelines for Americans for aerobic activity and time spent on sedentary behavior among US adults, 2007 to 2016. JAMA Netw open.

[4] Hovell MF, Wahlgren DR, Adams MA. The logical and empirical basis for the Behavioral Ecological Model. In *Emerging Theories in Health Promotion Practice and Research. Volume* 2nd. Edited by DiClemente RJ. San Francisco: Jossey-Bass; 2009.

[5] Sallis JF, Owen N, Fisher E. Ecological models of health behavior. In *Health Behavior and Health Education: Theory, Research, and Practice. Volume* 4th. Edited by Glanz K, Rimer BK, Viswanath K. San Francisco: Jossey-Bass; 2009: 465–482.

[6] Sallis JF, Cervero RB, Ascher W, Henderson KA, Kraft MK, Kerr J (2006). An ecological approach to creating active living communities. Annu Rev Public Health.

[7] Brownson RC, Boehmer TK, Luke DA (2005). Declining rates of physical activity in the United States: what are the contributors?. Annu Rev Public Health.

[8] Frank LD, Schmid TL, Sallis JF, Chapman J, Saelens BE (2005). Linking objectively measured physical activity with objectively measured urban form: findings from SMARTRAQ. Am J Prev Med.

[9] Community Preventive Services Task Force. : Physical activity: Built environment approaches combining transportation system interventions with land use and environmental design. In *The Community Guide*; 2016.

[10] Timmermans EJ, Visser M, Wagtendonk AJ, Noordzij JM, Lakerveld J (2021). Associations of changes in neighbourhood walkability with changes in walking activity in older adults: a fixed effects analysis. BMC Public Health.

[11] McCormack GR, Patterson M, Frehlich L, Lorenzetti DL (2022). The association between the built environment and intervention-facilitated physical activity: a narrative systematic review. Int J Behav Nutr Phys Activity.

[12] Kerr J, Norman GJ, Adams MA, Ryan S, Frank L, Sallis JF, Calfas KJ, Patrick K (2010). Do neighborhood environments moderate the effect of physical activity lifestyle interventions in adults?. Health Place.

[13] Lee RE, Mama SK, Medina AV, Ho A, Adamus HJ (2012). Neighborhood factors influence physical activity among african american and hispanic or latina women. Health Place.

[14] Colom A, Mavoa S, Ruiz M, Wärnberg J, Muncunill J, Konieczna J, Vich G, Barón-López FJ, Fitó M, Salas-Salvadó J. Neighbourhood walkability and physical activity: moderating role of a physical activity intervention in overweight and obese older adults with metabolic syndrome. Age Ageing 2020. 10.1093/ageing/afaa246 PMC8248320 33219673

[15] McCormack GR, Spence JC, McHugh TL, Mummery WK (2022). The effect of neighborhood walkability on changes in physical activity and sedentary behavior during a 12-week pedometer-facilitated intervention. PLoS ONE.

[16] Zenk SN, Wilbur J, Wang E, McDevitt J, Oh A, Block R, McNeil S, Savar N (2009). Neighborhood environment and adherence to a walking intervention in african american women. Health Educ Behav.

[17] Hoehner CM, Handy SL, Yan Y, Blair SN, Berrigan D (2011). Association between neighborhood walkability, cardiorespiratory fitness and body-mass index. Soc Sci Med.

[18] Sugiyama T, Shibata A, Koobsan MJ, Tanamas SK, Oka K, Salmon J, Dustan DW, Owen N. Neighborhood Environmental Attributes and Adults’ Maintenance of Regular Walking. *Medicine and Science in Sports and Exercise* in press. 10.1249/MSS.0000000000000528 25251048

[19] Riley DL, Mark AE, Kristjansson E, Sawada MC, Reid RD (2013). Neighbourhood walkability and physical activity among family members of people with heart disease who participated in a randomized controlled trial of a behavioural risk reduction intervention. Health Place.

[20] Frank LD, Sallis JF, Saelens BE, et al. The development of a walkability index: application to the Neighborhood Quality of Life Study. British Journal of Sports Medicine 2010;44:924–933. 10.1136/bjsm.2009.058701 19406732

[21] Adams MA, Todd M, Angadi SS, Hurley JC, Stecher C, Berardi V, Phillips CB, McEntee ML, Hovell MF, Hooker SP (2022). Adaptive goals and reinforcement timing to increase physical activity in adults: a Factorial Randomized Trial. Am J Prev Med.

[22] Vandelanotte C, Muller AM, Short CE, Hingle M, Nathan N, Williams SL, Lopez ML, Parekh S, Maher CA (2016). Past, Present, and future of eHealth and mHealth Research to improve physical activity and dietary behaviors. J Nutr Educ Behav.

[23] Adams MA, Hurley JC, Phillips CB, Todd M, Angadi SS, Berardi V, Hovell MF, Hooker S (2019). Rationale, design, and baseline characteristics of WalkIT Arizona: a factorial randomized trial testing adaptive goals and financial reinforcement to increase walking across higher and lower walkable neighborhoods. Contemp Clin Trials.

[24] U.S. Department of Health and Human Services. 2008 physical activity guidelines for Americans. U.S. Department of Health and Human Services; 2008.

[25] Adams MA, Hurley JC, Todd M, Bhuiyan N, Jarrett CL, Tucker WJ, Hollingshead KE, Angadi SS (2017). Adaptive goal setting and financial incentives: a 2 × 2 factorial randomized controlled trial to increase adults’ physical activity. BMC Public Health.

[26] Adams MA, Sallis JF, Norman GJ, Hovell MF, Hekler EB, Perata E (2013). An adaptive physical activity intervention for overweight adults: a randomized controlled trial. PLoS ONE.

[27] Adams MA, Caparosa S, Thompson S, Norman GJ (2009). Translating physical activity recommendations for overweight adolescents to steps per day. Am J Prev Med.

[28] Choi L, Ward SC, Schnelle JF, Buchowski MS (2012). Assessment of wear/nonwear time classification algorithms for triaxial accelerometer. Med Sci Sports Exerc.

[29] Adams MA, Frank LD, Schipperijn J, Smith G, Chapman J, Christiansen LB, Coffee N, Salvo D, du Toit L, Dygryn J (2014). International variation in neighborhood walkability, transit, and recreation environments using geographic information systems: the IPEN adult study. Int J Health Geogr.

[30] Brownson RC, Hoehner CM, Day K, Forsyth A, Sallis JF (2009). Measuring the built environment for physical activity: state of the science. Am J Prev Med.

[31] James P, Berrigan D, Hart JE, Hipp JA, Hoehner CM, Kerr J, Major JM, Oka M, Laden F (2014). Effects of buffer size and shape on associations between the built environment and energy balance. Health Place.

[32] Brooks ME, Kristensen K, Van Benthem KJ, Magnusson A, Berg CW, Nielsen A, Skaug HJ, Machler M, Bolker BM (2017). glmmTMB balances speed and flexibility among packages for zero-inflated generalized linear mixed modeling. R J.

[33] Fox J. Effect displays in R for generalised linear models. Journal of Statistical Software. 2003;8:1–27.

[34] Fox, J. Weisberg, S. An R Companion to Applied Regression. 3rd Edition. Sage, Thousand Oaks, CA. 2019.

[35] Boeing G, Higgs C, Liu S, Giles-Corti B, Sallis JF, Cerin E, Lowe M, Adlakha D, Hinckson E, Moudon AV (2022). Using open data and open-source software to develop spatial indicators of urban design and transport features for achieving healthy and sustainable cities. Lancet Glob Health.

[36] Muller PO. Transportation and urban form: Stages in the spatial evolution of the American metropolis. In *The geography of urban transportation* 3rd edition. Edited by Hanson S, Giuliano G. New York: The Guilford Press; 2004: 59–85.

[37] Cain KL, Millstein RA, Sallis JF, Conway TL, Gavand KA, Frank LD, Saelens BE, Geremia CM, Chapman J, Adams MA (2014). Contribution of streetscape audits to explanation of physical activity in four age groups based on the Microscale audit of Pedestrian Streetscapes (MAPS). Soc Sci Med.

[38] Prochnow T, Curran LS, Amo C, Patterson MS. Bridging the built and Social environments: a systematic review of studies investigating influences on physical activity. J Phys Act Health 2023:1–22. 10.1123/jpah.2022-0403 36997160

[39] Foster S, Giles-Corti B (2008). The built environment, neighborhood crime and constrained physical activity: an exploration of inconsistent findings. Prev Med.

