# Supplementary material for: The moderating impact of neighborhood walkability on mHealth interventions to increase moderate to vigorous physical activity for insufficiently active adults in a randomized trial
Source: Int J Behav Nutr Phys Act. 2023 Aug 15;20:97. doi: 10.1186/s12966-023-01494-2 (PMC10428579; doi:10.1186/s12966-023-01494-2)
Supplement: Supplementary file 3 — Supplementary Material 3: Appendix Figure 1. Walkability Index scores for participants’ 500-m network buffers (N=512). [file 12966_2023_1494_MOESM3_ESM.docx]

Appendix Figure 1. Walkability Index scores for participants’ 500-m network buffers (N=512).


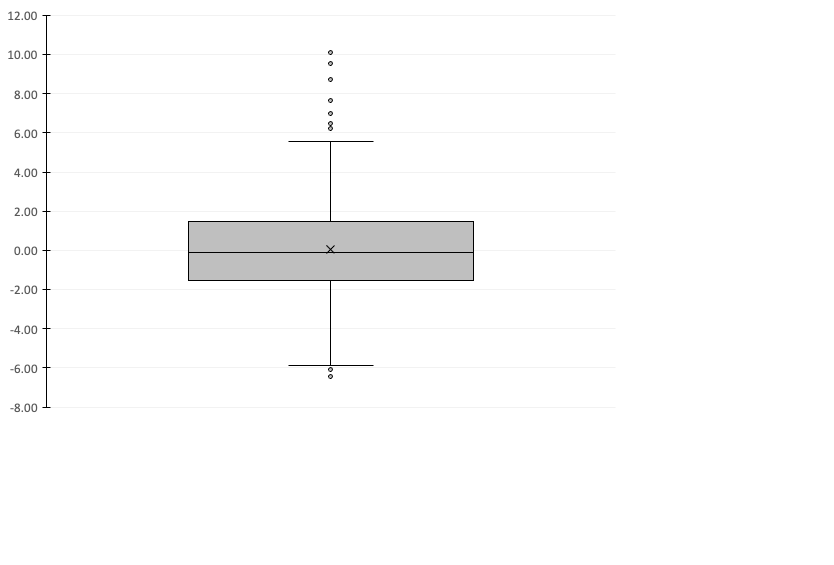


500-m Walkability Index^1^

(z-scores)

^1^Circles are outliers that extend past the whiskers and the “X” represents the mean in this box-and-whisker plot. The Walkability Index z-score equaled the sum of z-scores for residential density, land use mix, transit, and intersection densities. Z-scores allowed for standardized values necessary for comparisons across components.
